# Supplementary material for: A Comprehensive Assessment of Ultraviolet-Radiation-Induced Mutations in Flammulina filiformis Using Whole-Genome Resequencing
Source: J Fungi (Basel). 2024 Mar 20;10(3):228. doi: 10.3390/jof10030228 (PMC10971301; doi:10.3390/jof10030228)
Supplement: Supplementary file 1 [file jof-10-00228-s001.zip › Supplementary Material S8/KEGG annotation/out/64381550635650.os/KO/out_map/map05212.html]

KEGG PATHWAY: Pancreatic cancer - Reference pathway


|  |  |
| --- | --- |
| **Pancreatic cancer - Reference pathway** |  |

[
Pathway menu
| Organism menu
| Pathway entry
| Show description
| User data mapping
]

Infiltrating ductal adenocarcinoma is the most common malignancy of the pancreas. When most investigators use the term 'pancreatic cancer' they are referring to pancreatic ductal adenocarcinoma (PDA). Normal duct epithelium progresses to infiltrating cancer through a series of histologically defined precursors (PanINs). The overexpression of HER-2/neu and activating point mutations in the K-ras gene occur early, inactivation of the p16 gene at an intermediate stage, and the inactivation of p53, SMAD4, and BRCA2 occur relatively late. Activated K-ras engages multiple effector pathways. Although EGF receptors are conventionally regarded as upstream activators of RAS proteins, they can also act as RAS signal transducers via RAS-induced autocrine activation of the EGFR family ligands. Moreover, PDA shows extensive genomic instability and aneuploidy. Telomere attrition and mutations in p53 and BRCA2 are likely to contribute to these phenotypes. Inactivation of the SMAD4 tumour suppressor gene leads to loss of the inhibitory influence of the transforming growth factor-beta signalling pathway.

|  |  |
| --- | --- |
| Reference pathway | 100% |
